# Quantitative trait loci identification, fine mapping and gene expression profiling for ovicidal response to whitebacked planthopper (*Sogatella furcifera* Horvath) in rice (*Oryza sativa* L.)

**DOI:** 10.1186/1471-2229-14-145

**Published:** 2014-05-28

**Authors:** Yaolong Yang, Jie Xu, Yujia Leng, Guosheng Xiong, Jiang Hu, Guangheng Zhang, Lichao Huang, Lan Wang, Longbiao Guo, Jiayang Li, Feng Chen, Qian Qian, Dali Zeng

**Affiliations:** 1 State Key Lab for Rice Biology, China National Rice Research Institute, Hangzhou 310006, P. R. China; 2 Institute of Genetics and Developmental Biology, Chinese Academy of Sciences, Beijing 100101, China; 3 Department of Plant Sciences, University of Tennessee, Knoxville, TN 37996, USA

**Keywords:** Fine mapping, Gene expression profiling, Ovicidal response, *qWL6*, *Sogatella furcifera* Horváth, Whitebacked planthopper

## Abstract

**Background:**

The whitebacked planthopper (WBPH), *Sogatella furcifera* Horváth, is a serious rice pest in Asia. Ovicidal resistance is a natural rice defense mechanism against WBPH and is characterized by the formation of watery lesions (WLs) and increased egg mortality (EM) at the WBPH oviposition sites.

**Results:**

This study aimed to understand the genetic and molecular basis of rice ovicidal resistance to WBPH by combining genetic and genomic analyses. First, the ovicidal trait in doubled haploid rice lines derived from a WBPH-resistant cultivar (CJ06) and a WBPH-susceptible cultivar (TN1) were phenotyped based on the necrotic symptoms of the leaf sheaths and EM. Using a constructed molecular linkage map, 19 quantitative trait loci (QTLs) associated with WLs and EM were identified on eight chromosomes. Of them, *qWL6* was determined to be a major QTL for WL. Based on chromosome segment substitution lines and a residual heterozygous population, a high-resolution linkage analysis further defined the *qWL6* locus to a 122-kb region on chromosome 6, which was annotated to encode 20 candidate genes. We then conducted an Affymetrix microarray analysis to determine the transcript abundance in the CJ06 and TN1 plants. Upon WBPH infestation, 432 genes in CJ06 and 257 genes in TN1 were significantly up-regulated, while 802 genes in CJ06 and 398 genes in TN1 were significantly down-regulated. This suggests that remarkable global changes in gene expression contribute to the ovicidal resistance of rice. Notably, four genes in the 122-kb region of the *qWL6* locus were differentially regulated between CJ06 and TN1 in response to the WBPH infestation, suggesting they may be candidate resistance genes.

**Conclusions:**

The information obtained from the fine mapping of *qWL6* and the microarray analyses will facilitate the isolation of this important resistance gene and its use in breeding WBPH-resistant rice.

## Background

Rice (*Oryza sativa* L.) is one of the world’s most important crops, providing a staple food for nearly half of the global population. In Asia, Africa, and Latin America, the demand for rice is expected to increase due to the steadily increasing population [1]. In China, for example, rice production will need to increase by approximately 20% by 2030 to meet the domestic demand if rice consumption per capita remains at its current level [2]. Yet rice production is continually threatened by insects, diseases, and other stresses. In recent years, rice infestations by insects have intensified across Asia, resulting in heavy yield losses [3].

The whitebacked planthopper (WBPH), *Sogatella furcifera* Horváth, is a serious rice pest in Asia. It damages the plants by sucking sap from the phloem and transmitting viruses, which lead to reductions in plant height, number of productive tillers, filled grains, and yield [4,5]. During the tillering stage, a heavy WBPH infestation results in the complete necrosis of rice plants, a condition commonly known as hopper burn [6-8]. The permanent breeding areas for the WBPH are in the tropics, where the population is maintained in the paddy field throughout the year. As an insect that can travel long distances, WBPH migrates from northern Vietnam to southern China, and then to central China and Japan, depending on the southwest monsoon in the rainy season. In temperate regions, WBPHs cannot live through the winter, and they are replaced each year by immigrants from southern regions [8]. In rice production practices, WBPH infestation is managed primarily by the use of chemical pesticides, which are both economically and environmentally costly. Moreover, the pesticides kill WBPH predators, and the overuse of pesticides prompts the evolution of resistance in the insects, which in turn leads to a pest resurgence. Some groups have produced rice plants transformed with *Bacillus thuringiensis* (*Bt*) genes for protection against WBPHs [9]. However, the potential ecological risks of transgenic plants may limit the deployment of Bt rice [10]. Thus, the exploitation of host plant resistance has generally been considered one of the most economical and environmentally friendly approaches for the management of WBPHs.

Classical genetic analysis of selected rice accessions has led to the identification of six major WBHP-resistance genes, *Wbph1* to *Wbph6 *[11]. *Wbph1* is located on the short arm of chromosome 7 near the RFLP marker, RG146A [12]. *Wbph2* is on the short arm of chromosome 6 in ARC10239 [13], *Wbph6* is on chromosome 11 and flanked by RM167 and RM267 [14]. The other three WBHP resistance genes, *Wbph3*–*5*, have not yet been mapped to the rice genetic map. In addition to these major WBPH-resistance genes, a number of quantitative trait loci (QTLs) have been identified that are associated with the quantitative resistance of rice to WBPHs. These QTLs were identified by analyzing various rice lines, including recombinant inbred lines (RILs) [8], doubled haploid (DH) populations [15], introgression lines using wild rice species as the resistance donor [16], and backcross-inbred lines (BILs) derived from interspecific crosses involving wild rice species [11]. Despite the lack of molecular identity for any of the WBPH resistance genes, some have been tentatively associated with either tolerance, antibiosis, or antixenosis, the three types of natural rice resistance mechanisms against WBPH [17].

One type of rice antibiosis resistance to WBPHs is ovicidal resistance, which is characterized by the formation of watery lesions (WLs) that result in the death of the WBPH eggs at those sites within 12 h of oviposition [18]. The egg mortality (EM) depends on the rice developmental stage and is greatest at the maximum tillering stage. This ovicidal response to WBPHs is especially prominent in the *japonica* cultivars in Japan [8]. In addition, Seino et al. (1996) found that benzyl benzoate was present in the watery lesions of some *japonica* rice, but was undetectable in the intact plant tissue and non-watery lesions [19], suggesting benzyl benzoate was the ovicidal substance in the watery lesions.

Regarding the genetic basis of the rice ovicidal response to WBPHs, a total of 15 QTLs have been identified using the rice RILs developed from a cross between the WBPH-resistant *japonica* variety Asominori and the WBPH-susceptible *indica* variety IR24 [8]. Four of the 15 QTLs were further shown to be for the ovicidal trait based on the phenotyping for EM [4]. Nevertheless, our understanding of the genetic basis of WL induction for WBPH resistance is extremely limited.

In addition to the continued identification of major resistance genes and QTLs, our general understanding of plant resistance to insect herbivory has significantly improved with the employment of various genomic tools, one of which is global gene expression profiling [20]. For rice, gene expression profiling has been performed to understand the defenses against chewing insects such as the fall armyworm [21], sap-sucking insect brown planthopper (BPH) [22], and water weevil [23]. These analyses showed that the defenses against these insects involved global changes in rice gene expression and led to the identification of a large number of candidate defense genes.

Our study of rice resistance to WBPHs has focused on the Chinese *japonica* rice variety Chunjiang 06 (CJ06), which showed the strongest ovicidal response to WBPHs among the rice lines screened [24]. In addition, CJ06 exhibited sucking-inhibitory resistance to the WBPH, a type of antixenosis resistance [25]. This dual-mechanism of WBPH resistance in CJ06 makes this variety a unique genetic material for studying rice resistance to WBPHs. Based on a cross between CJ06 and TN1, a WBPH-susceptible *indica* rice, we previously constructed a DH population containing 120 lines. Our previous characterization of the phenotypic expression of WBPH resistance in this DH population [26] suggested that the combined functions of both the major resistance genes and QTLs affected the host-plant response to infestations by WBPHs.

Building on our previous work, this study had two objectives. The first was to improve our understanding of the genetic basis of rice resistance to WBPHs. We aimed to identify the QTLs associated with the ovicidal response, especially those for WLs, using our CJ06/TN1 DH population. Once identified, these QTLs were mapped to the rice genetic maps through fine mapping. The second objective was to improve our understanding of the molecular basis of rice resistance to the WBPH. To this end, we conducted a microarray analysis to compare the gene expression changes in WBPH-infested and uninfested CJ06 and TN1 plants.

## Results

### TN1 and CJ06 exhibited distinct ovicidal responses to WBPH feeding

**Figure 1:**
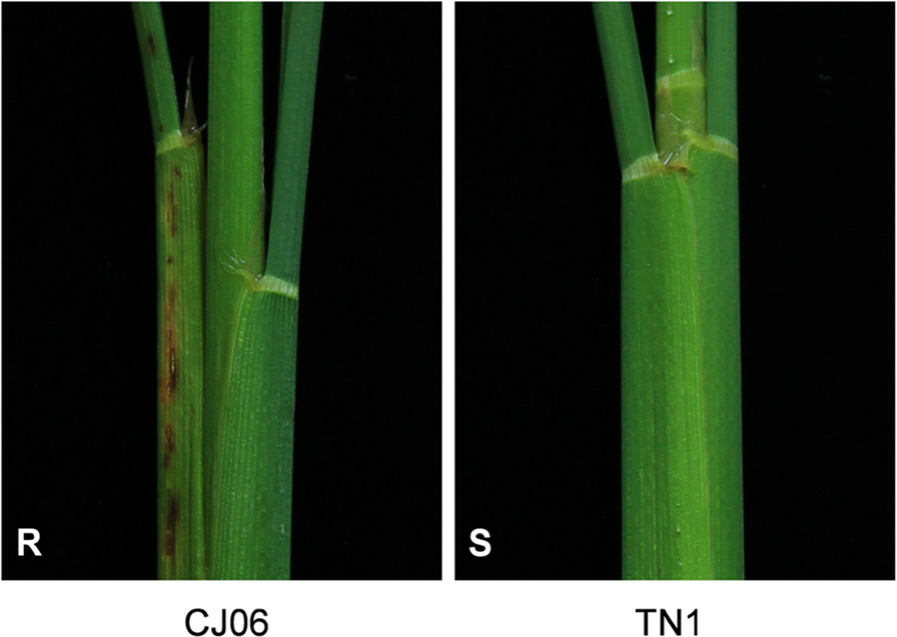
**Representative ovicidal response of the CJ06 and TN1 cultivars to whitebacked planthopper oviposition.** The leaf sheath of the resistant CJ06 variety was brownish black at the whitebacked planthopper oviposition sites and the watery lesions extended to the lateral veins around them. No visible symptoms appeared on the sheaths of the non-resistant TN1 variety.

CJ06, a *japonica* rice resistant to WBPHs, and TN1, an *indica* rice susceptible to WBPHs, exhibited pronounced differences in the necrotic discoloration of the leaf sheaths following oviposition by WBPHs (Figure 1). To quantify the differences in the ovicidal responses to WBPHs of these two rice varieties, the WLs were graded using a semi-quantitative scoring system (0, no visible necrotic symptoms; 1, brownish oviposition damage, but no watery lesions; 2, discontinuous watery lesions; and 3, conspicuous vertically elongated WLs) and the EM rates were determined (Table 1). These experiments were performed in two consecutive years, 2006 and 2007. The watery lesion grades for CJ06 were 2.6 and 2.8 in 2006 and 2007, respectively, whereas the TN1 WL grades were 0.2 and 0.4. The two varieties also showed significant differences in the WBPH-EM rates, with EM rates on CJ06 of 94.2% and 93.8% in 2006 and 2007, respectively, while those on TN1 were 19.2% and 11.8%.

**Table 1:** Ovicidal response in the parents and doubled haploid population

**Trait**		**Parents**		**DH population**	
		**CJ06**	**TN1**	**Means**	**deviation**
2006	WL (Score)	2.6 ± 0.55	0.2 ± 0.45**	1.30	0.119
	EM (%)	94.2 ± 13.0	19.2 ± 16.4**	58.4	-0.143
2007	WL (Score)	2.8 ± 0.45	0.4 ± 0.55**	1.12	1.161
	EM (%)	93.8 ± 10.8	11.8 ± 14.7**	60.0	-0.462

**t-test, significance at 0.01 level. WL, watery lesion; EM, egg mortality.

### The distribution of the ovicidal response in the DH population revealed a major locus and multiple minor loci for WBPH resistance

**Figure 2:**
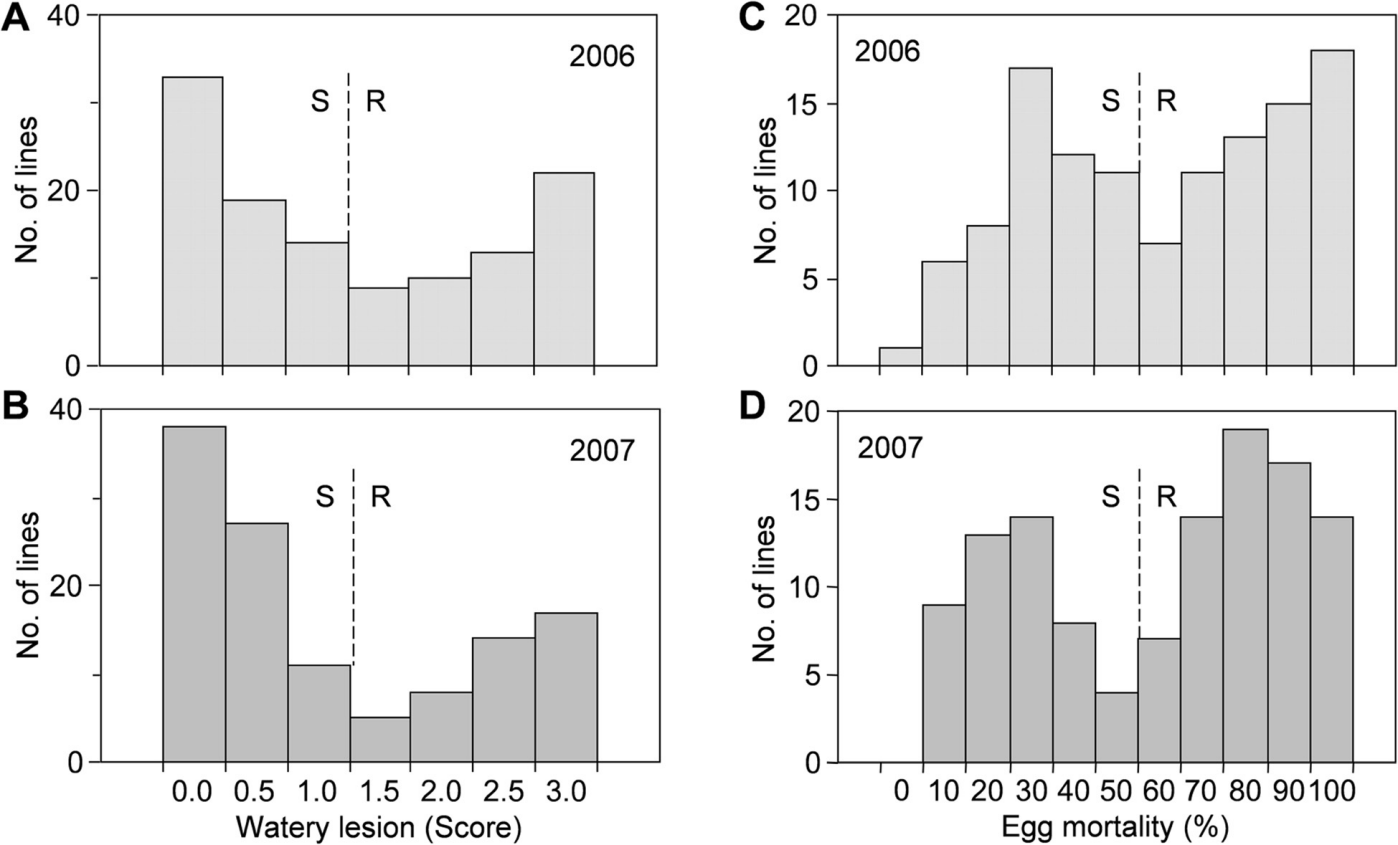
**Distribution of watery lesions and egg mortality in the doubled haploid population. (A)** and **(B)** show the distribution of watery lesions (WLs) in the doubled haploid populations in 2006 and 2007, respectively. **(C)** and **(D)** show the distribution of egg mortality. WL grading scheme: 0, no visible necrotic symptoms; 1, brownish oviposition damage, but no WLs; 2, discontinuous WLs; 3, conspicuous, vertically elongated WLs.

The WL and EM evaluations in the DH population derived from a CJ06 × TN1 cross were also executed in 2006 and 2007. The DH lines exhibited considerable quantitative variation for these WBPH-resistance traits (Figure 2). For the WLs, the DH lines had grades ranging from no visible symptoms (grade 0) to a very strong response (grade 3), with means of 1.30 and 1.12 in 2006 and 2007, respectively (Figure 2A and B, Table 1). Among the 120 DH lines, 66 demonstrated an ovicidal response, while 54 lines had non-ovicidal or slight responses, with nearly a 1:1 ratio of the two responses. Twenty-two of the lines expressed a strong ovicidal response (grade 3) in 2006, while 33 had no response (grade 0). In 2007, 72 and 48 lines were identified as ovicidal and non-ovicidal, respectively. The WBPH-EM rate on the DH lines ranged from 0–100%, with means of 58.4% and 60.0% in 2006 and 2007, respectively (Figure 2C and D, Table 1). In 2006, 64 lines resulted in high EM rates, which was low 55 other lines. Similarly in 2007, 71 and 48 lines were classified as producing high and low EM, respectively. The resistance levels of some of the DH lines exceeded those of the parents, which indicated the presence of transgressive variation for the ovicidal response to WBPHs. The frequency distribution of the resistant-trait phenotypes for the WL and EM in the DH lines clearly displayed the spectrum of one major locus and multiple minor loci for WBPH resistance in the DH population.

### The detection of quantitative trait loci associated with watery lesions and egg mortality

**Figure 3:**
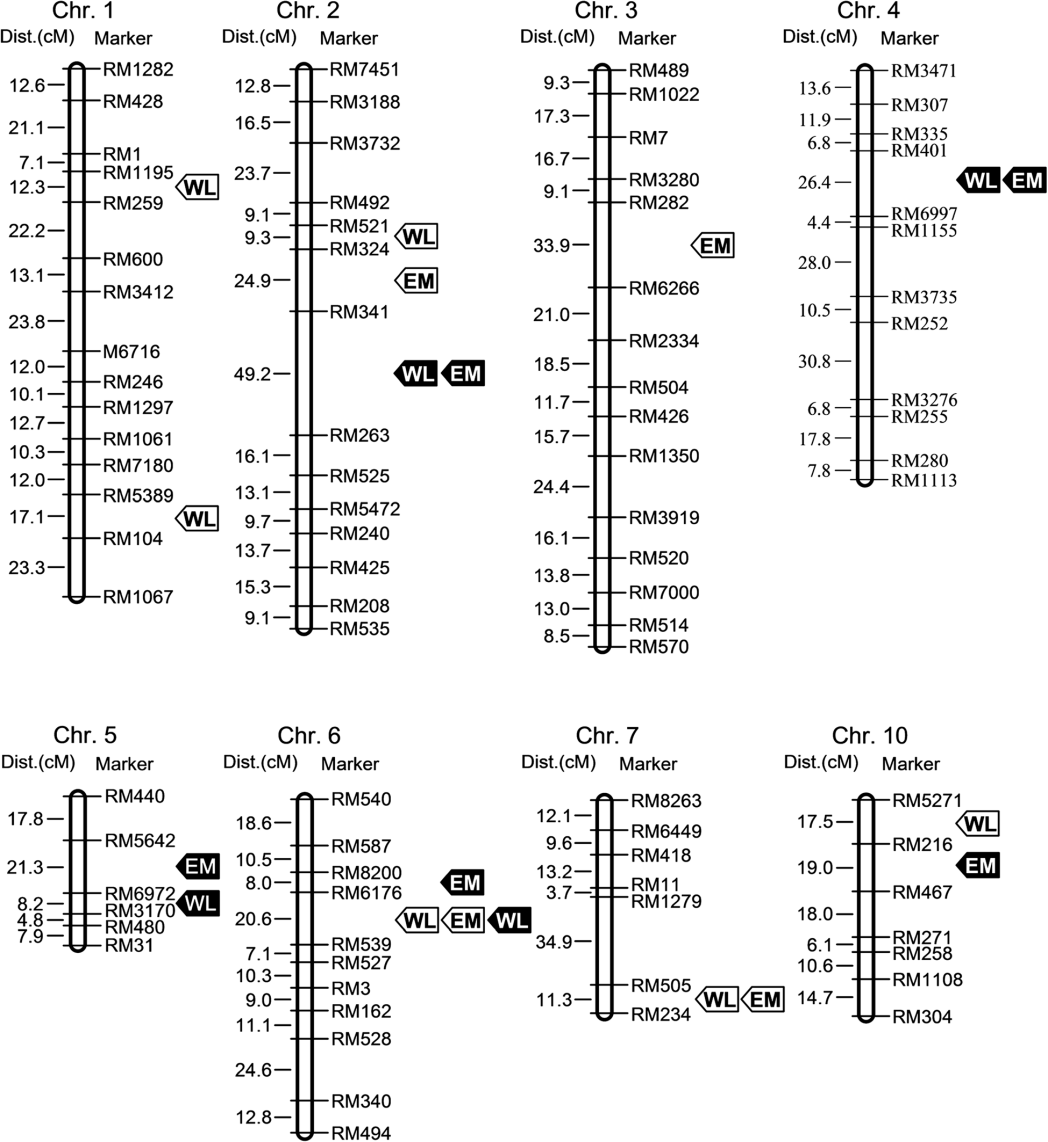
**Chromosomal locations of the putative ovicidal response quantitative trait loci on the linkage map.** The map distances and marker names are shown on the left and right of the chromosome, respectively. Arrows indicate the peak LOD (logarithm of odds) positions of the putative quantitative trait loci (QTL) for the ovicidal response. Open and solid arrows indicate QTLs identified in 2006 and 2007, respectively.

To identify the genetic loci responsible for the ovicidal response, QTL analysis was performed by doing an association analysis for the WLs and EM with a molecular-marker linkage map, which was available for the CJ06/TN1 DH population. Ten QTLs associated with the WLs and EM were found and localized on six chromosomes in 2006, and 9 QTLs distributed on five chromosomes were identified based on the 2007 data (Figure 3, Table 2). Of these, some of the QTLs responsible for the WLs and EM were co-localized on the chromosomes, while other QTLs were found only in one of the two years. The QTLs associated with the WLs and EM near RM341 on chromosome 2 and close to RM6176 on chromosome 6 were consistently detected in both 2006 and 2007. However, the QTLs located on chromosomes 4 and 5 were only localized in 2007, and the loci on chromosome 7 were only found in 2006. QTLs associated with the ovicidal response were also observed on chromosomes 1, 3, and 10.

**Table 2:** Quantitative trait loci identified in the doubled haploid population for the ovicidal response

**Traits**	QTL*	**Chromosome**	**Interval**	**LOD**	**Variance explained (%)**	**Additive effect**
2006						
WL	*qWL1.1(1)*	1	RM575-RM259	2.72	6.79	0.53
	*qWL1.2(1)*	1	RM1198-RM104	3.31	13.78	-0.74
	*qWL2(1)*	2	RM521-RM324	3.09	15.61	-0.84
	*qWL6*	6	RM6176-RM539	12.23	30.23	-1.67
	*qWL7(1)*	7	RM1279-RM505	2.81	8.51	-0.67
	*qWL10(1)*	10	RM5271-RM216	2.65	6.72	0.55
EM	*qEM2(1)*	2	RM324-RM341	3.01	16.74	-18.54
	*qEM3(1)*	3	RM282-RM6266	2.63	12.15	15.55
	*qEM6(1)*	6	RM6176-RM539	4.33	25.74	-31.45
	*qEM7(1)*	7	RM505-RM234	3.09	15.30	-17.83
*2007*						
WL	*qWL2(2)*	2	RM341-RM263	2.94	14.55	-0.78
	*qWL4(2)*	4	RM401-RM6997	2.47	9.13	0.78
	*qWL5(2)*	5	RM6972-RM3170	3.46	12.09	-0.8
	*qWL6*	6	RM6176-RM539	12.14	34.24	-1.73
EM	*qEM2(2)*	2	RM341-RM263	2.86	13.84	-19.85
	*qEM4(2)*	4	RM401-RM6997	2.31	9.06	14.03
	*qEM5(2)*	5	RM5642-RM6972	2.63	10.12	-16.05
	*qEM6(2)*	6	RM8200-RM6176	4.15	24.10	-34.88
	*qEM10(2)*	10	RM216-RM467	2.46	8.42	11.00

*The numbers “1” and “2” in brackets indicate the quantitative trait loci identified in 2006 and 2007, respectively.

Of the identified QTLs, most showed negative additive effects, suggesting the CJ06 alleles may increase the ovicidal response to WBPHs. The loci associated with the WLs that were flanked by RM6176 and RM539 on chromosome 6 presented the largest explained variance and showed additive effects. The LOD (Logarithm of Odds) scores were 12.23 and 12.14 in 2006 and 2007, respectively. Their proportion of the phenotypic variation was over 30%, with the CJ06 allele on chromosome 6 increasing the phenotypic grade for the WLs to approximately 1.6. This major QTL was named *qWL6*. The QTLs for WBPH EM were also found in this region both years, and the explained EM variation was nearly 25%. The CJ06 alleles on chromosomes 2, 5, and 7 and the long arm of chromosome 1 may strengthen the ovicidal response. We also found that some alleles from the TN1 variety may increase the resistance to WBPH, such as the loci on chromosomes 3, 4, and 10 and the short arm of chromosome 1.

### Development of the chromosome segment substitution lines for the major quantitative trait locus *qWL6*

**Figure 4:**
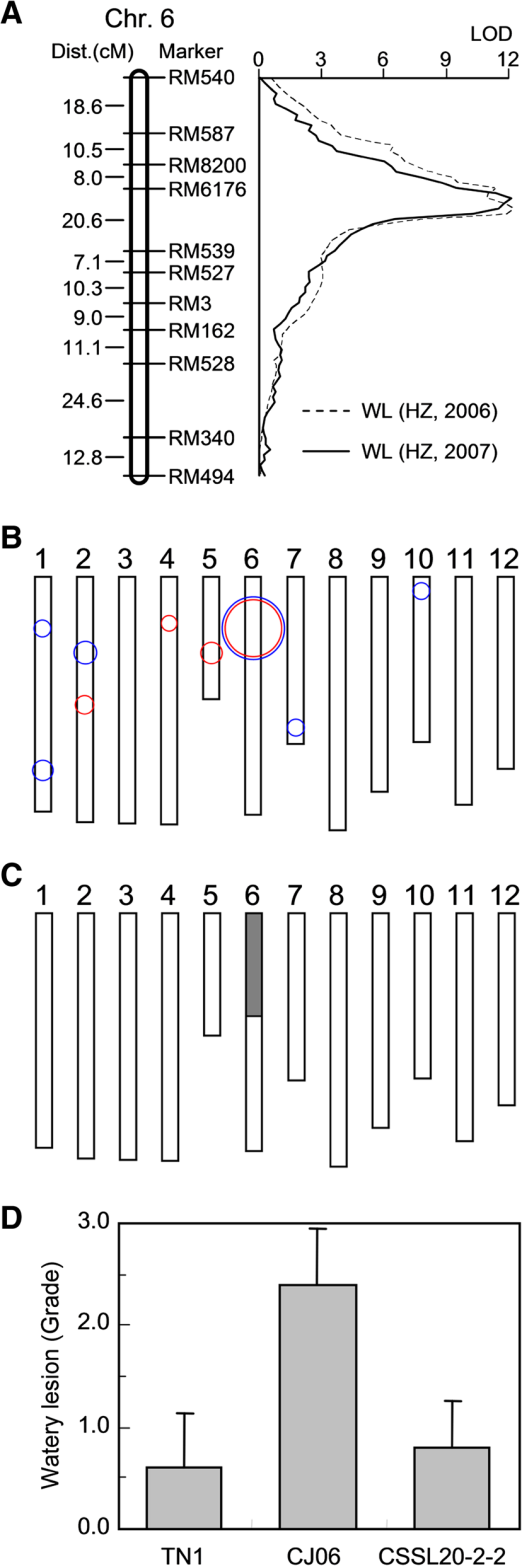
**Development of the chromosome segment substitution lines (CSSL). (A)** The genetic distance (Kosambi centiMorgan) and marker name are shown on the left and right of the chromosome, respectively. **(B)** Quantitative trait loci (QTL) analysis for watery lesions in the doubled haploid population. Circle centers indicate the positions of the QTLs on the rice chromosomes. Circle sizes indicate the LOD score for watery lesions. Blue and red circles indicate QTLs identified in 2006 and 2007, respectively. **(C)** Graphical genotype of CSSL20-2-2, a substitution line of chromosome 6. The black bar indicates the genome fragment from the non-resistant TN1variety; the other portions are from the resistant CJ06 variety. **(D)** Watery lesions in CJ06, TN1, and introgression CSSL20-2-2.

Based on the QTL analysis for WLs, the distribution of the LOD scores on chromosome 6 was determined (Figure 4A). There was a slight difference in the distribution curve between the two years, but the region of the maximum LOD score was similar under both years. Due to the difficulty of genetically transforming typical *indica* in the subsequent study, the *japonica* parent CJ06 was selected as the recurrent parent to introduce the susceptible *qWL6* allele from TN1. One line from the DH population was selected to cross with CJ06, followed by five successive backcrosses to CJ06. Simple sequence repeat (SSR) markers RM6176 and RM539 were used for the marker-assisted selection of the segregating progeny of each backcrossed generation. After five backcrosses with CJ06, the BC5F2 generation was scanned with a set of 74 SSR markers, which were uniformly distributed on the previous linkage map. One plant was selected, CSSL20-2-2, which carried a homozygous introgression from TN1 across the entire *qWL6* region in the CJ06 genetic background and was devoid of other QTLs in the region (Figure 4B and C). To confirm the phenotype of this line, WL production in response to WBPHs was investigated. The grade of WLs on CSSL20-2-2 was dramatically reduced in comparison to its CJ06 recurrent parent and was similar to that of TN1 (Figure 4D).

### Fine mapping of *qWL6* to a 122-kb region

**Figure 5:**
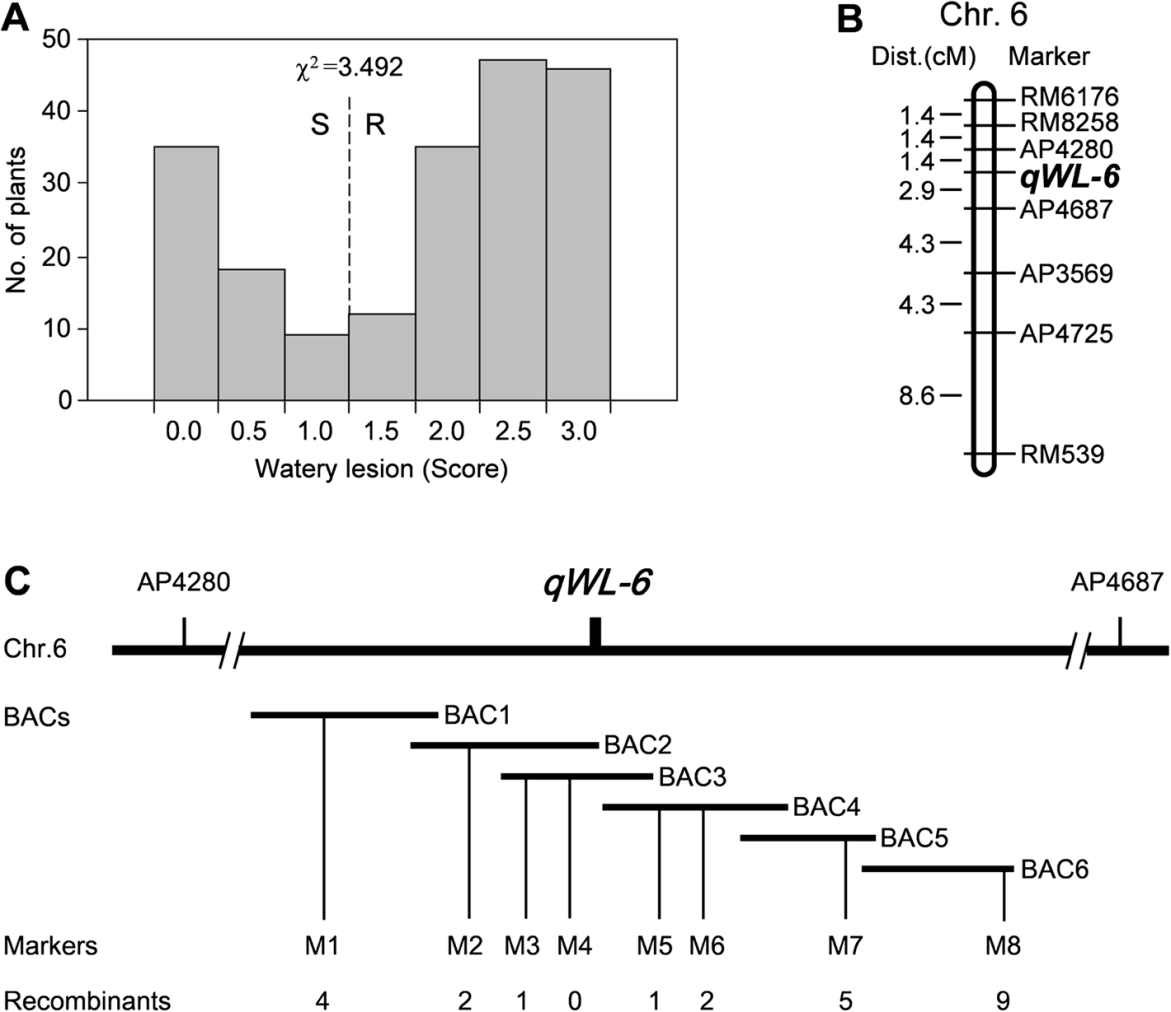
**Fine mapping of the major quantitative trait locus *qWL6*. (A)** The phenotypic values for the watery lesions (WLs) of each plant selected from the BC5F2 generation; 202 of the selected plants showed discontinuous and bimodal distributions for the WLs, with one low scoring region (no or slight ovicidal response) and another high scoring region (strong ovicidal response). **(B)** High density molecular-linkage map of rice chromosome 6 showing the location of *qWL6*. The genetic distance (Kosambi centiMorgan) and marker name are shown on the left and right of the chromosome, respectively. **(C)** The *qWL6* locus was mapped to the short arm of chromosome 6 between markers AP4280 and AP4687. Several BAC contig spanned the *qWL6* locus. The numerals indicate the number of recombinants identified from the BC5F2 mutant plants. BAC1, P0528E04; BAC2, B1172G12; BAC3, OJ1147_D11; BAC4, OSJNBa0016O19; BAC 5, OSJNBb0015B15; BAC 6, P0529B09.

The segregation population derived from a residual heterozygous line in the BC5F2 generation with heterozygosity only in the *qWL6* region was used for fine mapping (Figure 4C). This population displayed a clear bimodal distribution for WLs and was classified into ovicidal and non-ovicidal response groups (Figure 5A). Among these 202 plants, 35 had no visible response and 46 plants showed strong ovicidal responses. Based on the necrotic ovicidal symptoms, the 62 plants showing no visible response or only brownish oviposition damage were marked as not having ovicidal resistance, and the 140 plants with moderate to conspicuous watery lesions were designated as having ovicidal resistance. This segregation ratio fits the expected 3:1 ratio for single dominant gene segregation (χ^2^ = 3.492), suggesting that a single dominant gene derived from CJ06 caused the strong WL response. The 35 plants without visible WLs were used for further gene mapping. Eight developed Indel (insertion/deletion) markers and two SSR markers were selected to scan for polymorphisms between CJ06 and TN1, and five of the markers (RM8258, AP4280, AP4687, AP3569, and AP6056) were polymorphic between the two parents. Based on these results, a regional linkage map of *qWL6* was constructed (Figure 5B). RM6176, RM8258, and AP4280 were determined to be 4.2 cM, 2.8 cM and 1.4 cM from *qWL6*, respectively, on one side; RM539, AP4725, AP3569, and AP4687 were determined to be 20.1 cM, 11.5 cM, 7.2 cM, and 2.9 cM from *qWL6*, respectively, on the other side. The BC5F3 seeds of the 35 plants without visible WLs were harvested for further [27] analysis of their ovicidal resistance in the F3 progeny. None of them showed ovicidal responses after oviposition by WBPH (data not shown).

For fine mapping of the *qWL6* gene, the SSR marker RM8258 on one side of the *qWL6* target region and the Indel marker AP4687 on the other side were used to identify recombination break points in the segregating progeny derived from the residual heterozygous lines. Seedlings (n = 216) selected from 1,440 BC6F2 progeny were transplanted into greenhouse conditions to evaluate their ovicidal resistance to WBPHs. Of these, 41 displayed no visible WLs and were used for further fine mapping. The analysis of RM8258 identified 10 recombination events between it and *qWL6* on one side, and the analysis of AP4687 detected 31 recombination events between the Indel marker and *qWL6* on the other side. Eight polymorphic markers were available to narrow down the region of the *qWL6* locus. AP4280 detected 7 recombinants, whereas Indel marker M4 co-segregated with *qWL6*. The markers M1, M2, and M3 revealed 4, 2, and 1 recombinants, respectively, on one side, while markers M5, M6, M7, and M8 indicated 1, 2, 5, and 9 recombinants, respectively, on the other side (Figure 5C). Therefore, the *qWL6* locus was finally delimited to an approximately 122.1-kb DNA region between the two Indel markers, M3 and M5.

This 122.1-kb region of the Nipponbare rice genome retrieved from the Rice Genome Annotation Project database (http://rice.plantbiology.msu.edu/annotation_pseudo_current.shtml) encoded 20 genes in its annotation (gene IDs from LOC_Os06g09910 to LOC_Os06g10109). Two candidate genes, LOC_Os06g09910 and LOC_Os06g09930, were predicted to be phosphopantothenoylcysteine decarboxylase (PPCDC) and a G protein coupled receptor (GPCR), respectively; the others are unknown proteins.

### Differentially expressed genes between CJ06 and TN1

**Figure 6:**
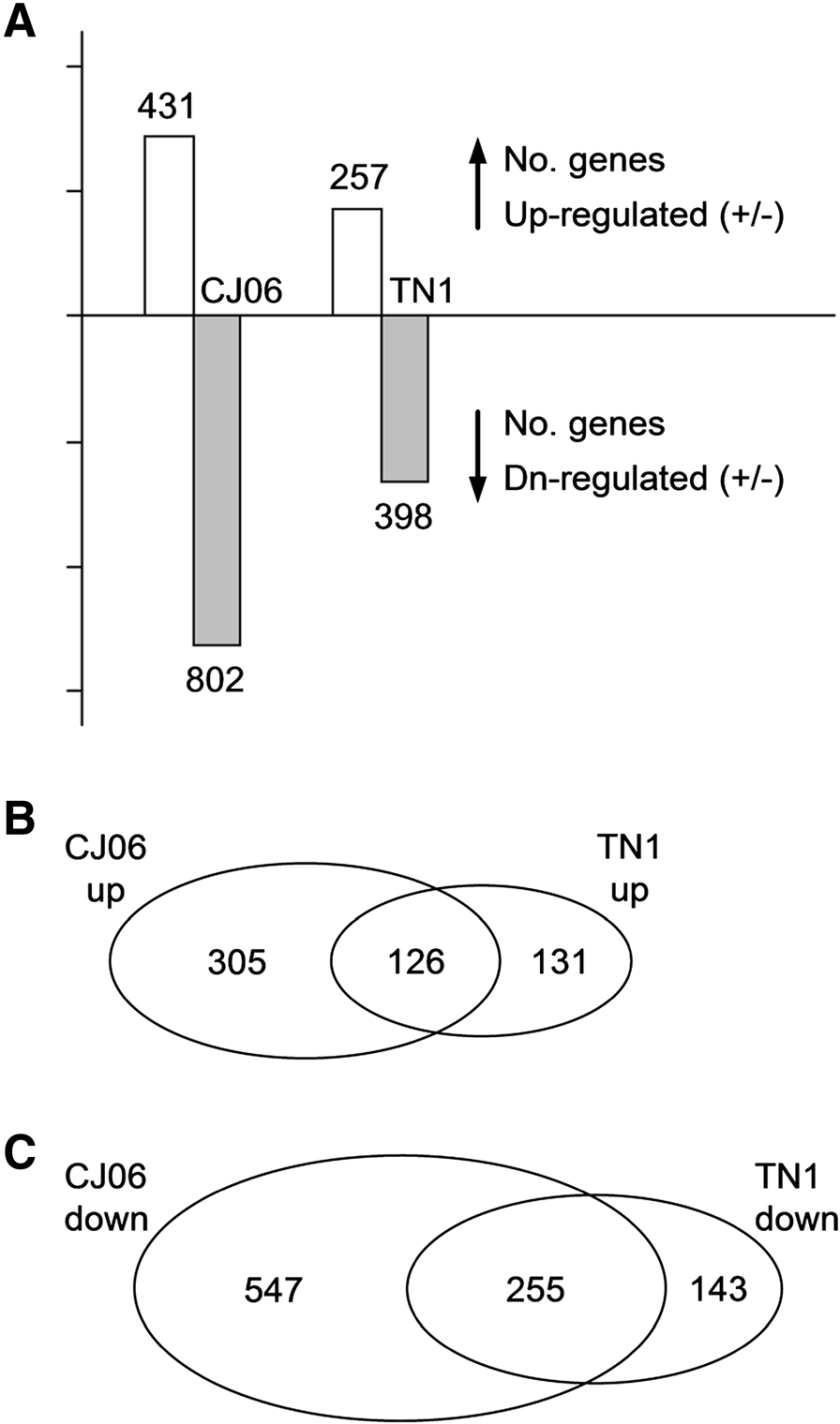
**Number of differentially expressed probe sets. (A)** The histogram shows the total number of probe sets that were up- or down-regulated two-fold or more in the resistant CJ06 and non-resistant TN1 varieties in response to whitebacked planthopper feeding. Venn diagrams illustrate the number of probe sets up-regulated **(B)** and down-regulated **(C)** during a whitebacked planthopper infestation.

To further analyze the molecular mechanism underlying rice WBPH resistance, whole genome transcript profiling using Affymetrix microarrays was performed to examine the expression levels of all of the rice genes in infested and uninfested CJ06 and TN1 plants. Three-fold changes were used as a threshold to judge significantly different expression.In WBPH-infested CJ06, 431 and 802 genes were found to be significantly up-regulated and down-regulated, respectively. In contrast, the numbers of significantly up-regulated and down-regulated genes induced by the WPPH infestation in TN1 were 257 and 398, respectively (Figure 6A). Among the up-regulated genes in response to the WBPH infestation in the two varieties, 126 were shared (Figure 6B), while the number of genes that were down-regulated in both varieties was 255 (Figure 6C).

**Figure 7:**
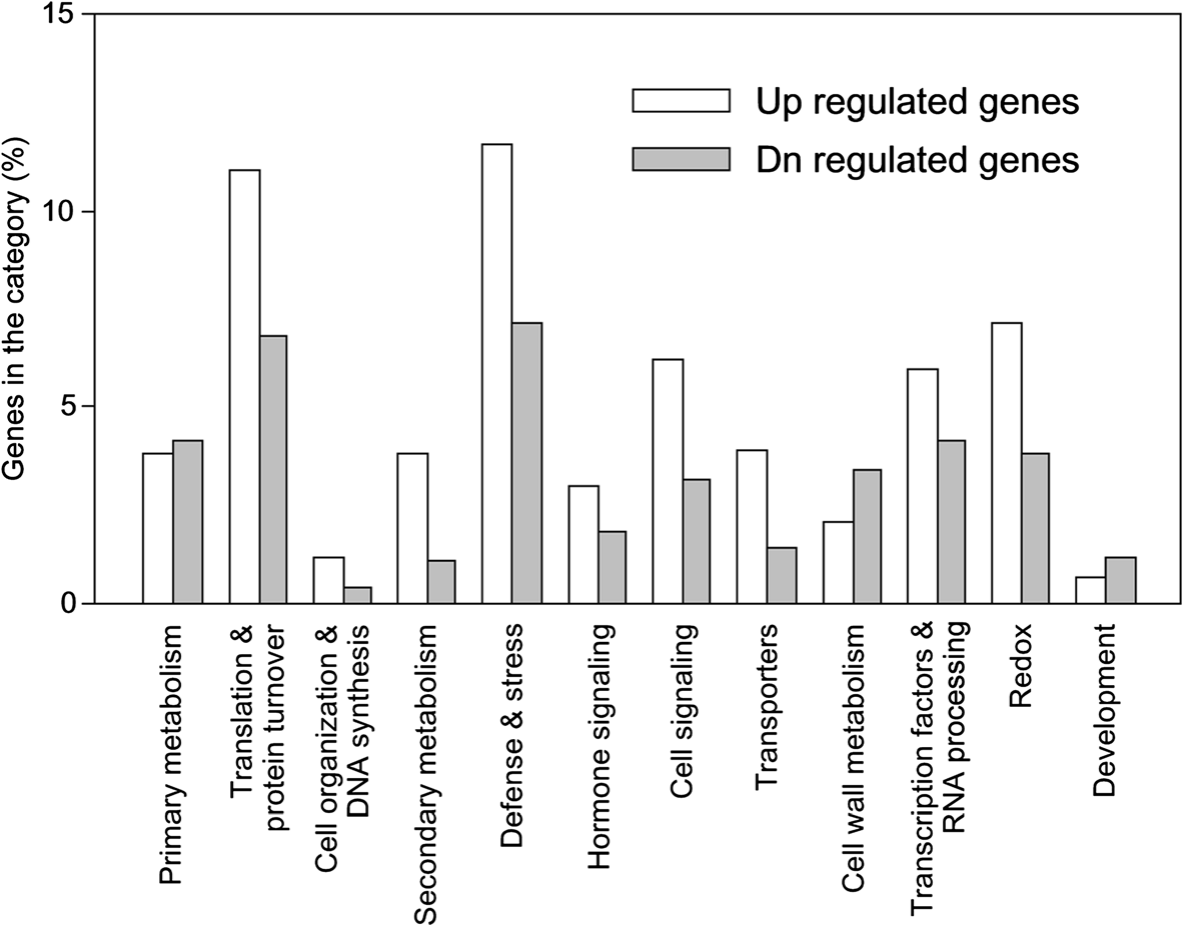
**Biological functional classification of the differentially expressed genes between the CJ06 and TN1 varieties.** A combined criterion of a 3-fold difference in expression was used, which resulted in 760 genes being identified as differentially expressed. White columns: 465 genes with more transcripts in the resistant CJ06 variety than in the non-resistant TN1 variety; dark columns: 295 genes with fewer transcripts in CJ06 than in TN1.

To gain an understanding of the basis of defense, comparisons of gene expression were also made between the CJ06 and TN1 plants. A total of 760 genes were differentially expressed and further assigned to different functional categories (Figure 7, Additional file 1). The differentially expressed genes related to secondary metabolism, defense, transport, translation, and protein turnover were overrepresented in CJ06. For instance, among the 35 differentially expressed genes for secondary metabolism, 29 had high levels of expression in CJ06, whereas only 9 showed high levels of expression in TN1.

Some of the differentially expressed genes in CJ06 and TN1 are listed in Table 3. Genes related to defense, secondary metabolism, transcription, and cell and hormone signaling were differentially expressed between CJ06 and TN1. Genes encoding pathogenesis-related protein, germin-like protein, thaumatin-like protein, and α-amylase/trypsin inhibitor were drastically up-regulated in CJ06. After feeding by WBPH, genes involved in secondary metabolism such as terpene synthase, anthranilate N-benzoyltransferase, agmatine coumaroyltransferase, and multicopper oxidase family protein were intensively up-regulated. Genes pertaining to cell signaling and RNA processing and transcription also showed differentially induced expression. In contrast, genes implicated in hormone signaling such as auxin-responsive protein, VQ motif protein, and ARR8 protein were strongly suppressed in CJ06. Surprisingly, the genes that were highly differentially expressed were not located on chromosome 6, where the major QTL for the ovicidal response was observed. Comparing the sequences of some of the candidate genes in the 122-kb region, four of the six completely sequenced candidate genes had putative nonsynonymous substitutions (Table 4). A functional difference based on the diversity of the amino acid sequences may also play an important role in WBPH resistance.

**Table 3:** Genes differentially expressed in the whitebacked planthopper-infested CJ06 and TN1 varieties

**Putative function**	**Probe set ID**	**Locus ID**	**Fold-change**		
			**CJ(+/-)**^**a**^	**TN(+/-)**^**b**^	**(CJ+/TN+)**^**c**^
**Defense and stress related genes**					
Pathogenesis-related thaumatin-like protein	Os.49615.1.S1_at	LOC_Os03g45960	15.26	5.91	5.31
Germin-like protein subfamily 1 precursor	Os.47320.1.A1_at	LOC_Os04g52720	19.24	6.31	2.90
Pathogenesis-related protein precursor	Os.51641.1.S1_x_at	LOC_Os07g03590	19.50	9.29	2.74
Pathogenesis-related protein PRB1precursor	Os.9421.1.S1_at	LOC_Os10g11500	50.58	7.40	6.99
Alpha-amylase/trypsin inhibitor	OsAffx.32171.3.S1_at	LOC_Os12g43490	6.93	2.02	19.50
**Secondary metabolism**					
Terpene synthase	OsAffx.25825.1.S1_at	LOC_Os04g01810	7.61	2.86	2.28
Anthranilate N-benzoyltransferase protein 1	Os.49568.1.S1_at	LOC_Os04g56900	17.08	2.05	6.04
Agmatine coumaroyltransferase	Os.51179.1.S1_at	LOC_Os11g42290	6.58	2.29	2.36
Multicopper oxidase family protein	Os.17659.1.S1_at	LOC_Os12g15680	14.46	3.45	4.16
**Genes involved in cell signaling**					
S-locus-like receptor protein kinase	Os.48003.1.A1_at	LOC_Os01g47840	8.73	2.79	2.70
ATP binding protein	Os.26226.1.S1_at	LOC_Os02g02780	7.77	2.35	2.33
TAK14	Os.778.2.S1_x_at	LOC_Os01g02700	2.60	-2.32	2.47
Leucine Rich Repeat family protein	OsAffx.20258.1.S1_at	LOC_Os10g33080	2.86	-2.03	5.25
**Transcription factors and genes involved in RNA processing**					
NAC domain-containing protein 18	Os.802.1.S1_at	LOC_Os01g01430	6.10	2.08	2.62
Myb-like DNA-binding domain containing	Os.31381.1.S1_at	LOC_Os01g03720	5.26	2.02	2.78
Helix-loop-helix DNA-binding domain	Os.32770.1.S1_x_at	LOC_Os01g09930	7.10	2.79	3.19
NAC-domain containing protein 90	Os.34471.1.S1_at	LOC_Os01g64310	2.72	-2.04	3.92
Transcription factor MYC7E	Os.46443.1.S1_at	LOC_Os10g42430	2.55	-2.33	2.16
**Genes implicated in hormone signaling**					
Auxin efflux carrier component 4	Os.2230.1.S1_at	LOC_Os02g50960	-6.78	-2.05	-3.34
Auxin-responsive protein IAA14	Os.8585.1.S1_at	LOC_Os03g53150	-6.79	-2.13	-2.03
Auxin responsive protein, expressed	Os.37213.1.S1_at	LOC_Os07g29310	-7.35	-3.07	-4.15
1-aminocyclopropane-1-carboxylate oxidase 1	Os.12201.2.S1_at	LOC_Os09g27820	-11.52	-4.11	-31.22
VQ motif family protein	Os.15204.1.S1_at	LOC_Os11g03660	-5.06	-2.14	-2.13
Two-component response regulator ARR8	Os.37430.1.S1_at	LOC_Os12g04500	-27.22	-4.40	-3.20

A combined criterion of a 3-fold or more change between CJ06 and TN1 was used to obtain genes with more than a 2-fold response to WBPH feeding. ^a^ratio of transcripts in WBPH-infested CJ06 to those in uninfested CJ06; ^b^ratio of transcripts in WBPH-infested TN1 to those in uninfested TN1; ^c^ratio of transcripts in the leaf sheaths of CJ06 to those in TN1 after feeding by WBPH. Positive and negative numerals indicate the gene expression in the infested leaf sheaths was up-regulated or down-regulated, respectively.

**Table 4:** Polymorphic sites of candidate genes and the putative amino acid differences between CJ06 and TN1

	**LOC_Os06g09910**		**LOC_Os06g09930**		**LOC_Os06g10000**	**LOC_Os06g10100**	
	+127	+580	+8	+3638	+101	+266	+773
CJ06	G	T	C	A	G	T	C
Putative AA	Ser	Phe	Ala	Ile	Ser	Phe	Ala
TN1	C	A	G	C	C	A	G
Putative AA	Thr	Tyr	Gly	Leu	Thr	Tyr	Gly

### Expression patterns of the candidate genes in the 122-kb region

**Figure 8:**
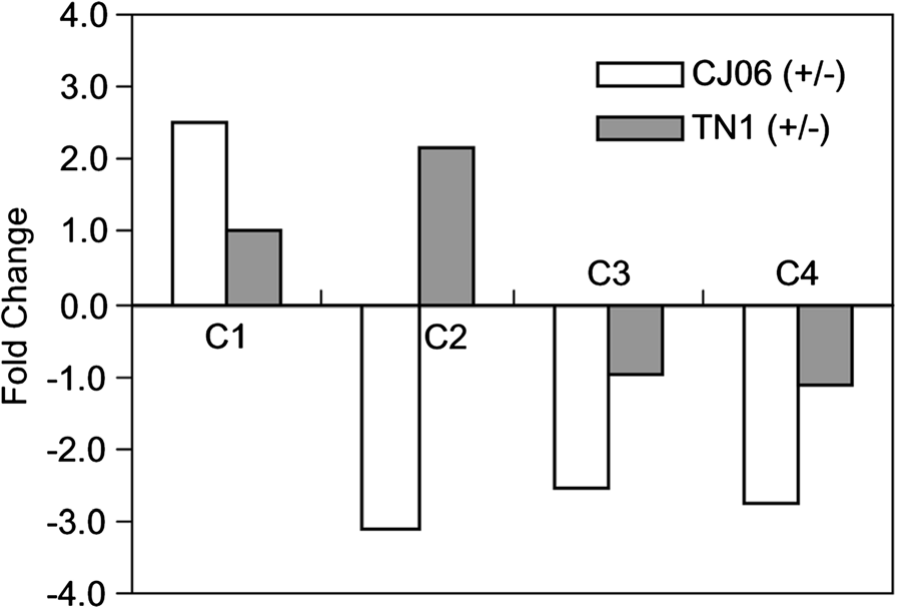
**Quantitative real-time PCR analysis of the expression of four candidate genes in CJ06 and TN1.** Values are the ratios of the transcript levels of each gene in whitebacked planthopper-infested leaf sheaths versus an uninfested control. C1–C4 indicate the candidate genes LOC_Os06g09960, LOC_Os06g09970, LOC_Os06g10000, and LOC_Os06g10109, respectively. Positive and negative numerals indicate the fold-change of the gene expression in the infested leaf sheaths was up-regulated or down-regulated, respectively.

Special attention was paid to the expression patterns of the candidate genes in the 122-kb region. The Affymetrix array contained probes for 15 of the 20 candidate genes (Table 5). While 11 of the genes showed no significant differences in expression, the remaining four (LOC_Os06g09960, LOC_Os06g09970, LOC_Os06g10000 and LOC_Os06g10109) were identified as differentially expressed between CJ06 and TN1 (Table 5). The transcript level of LOC_Os06g09960 was up-regulated 2-fold in CJ06 after feeding by WBPH, but did not change in TN1. The expression of LOC_Os06g09970 was suppressed in CJ06, but was up-regulated approximately 2-fold in TN1. The expression of LOC_Os06g10000 and LOC_Os06g10109 were down-regulated more than 2-fold in CJ06 under the WBPH infestation, while there were no evident changes in TN1.To confirm the divergence on the microarray was due to the diversity in the genetic background, the differential expression of these four genes were further validated using real-time reverse transcription polymerase chain reaction (RT-PCR) analysis (Figure 8). LOC_Os06g09960 was up-regulated approximately 3-fold in CJ06 during the WBPH infestation, whereas there was no distinct change in TN1. LOC_Os06g09970 was down-regulated more than 3-fold in infested CJ06 plants, yet it was up-regulated in TN1. LOC_Os06g10000 and LOC_Os06g10109 appeared to be down-regulated in CJ06 after the WBPH infestation, but there was no obvious change in expression in TN1 in response to the WBPH feeding. We also examined the expression of the other five candidate genes (LOC_Os06g09920, LOC_Os06g10010, LOC_Os06g10030, LOC_Os06g10050 and LOC_Os06g10090), but they did not display any differential expression (data not shown).

**Table 5:** Expression of candidate genes in the interval between the two Indel markers M3 and M5

**Gene ID**	**cDNA/ homologous EST**	**Probe Set ID**	**Putative function**	**Fold-change**	
				**CJ06(+/-)**^**a**^	**TN1(+/-)**^**b**^
LOC_Os06g09910	AK064613	Os.48661.1.S1_at	Phosphopantothenoylcysteine decarboxylase	-1.28	-1.04
LOC_Os06g09920	No	No	Expressed protein		
LOC_Os06g09930	AK111880	Os.39747.1.S1_at	G protein coupled receptor	-1.28	-1.19
LOC_Os06g09940	No	OsAffx.27540.1.S1_at	Expressed protein	1.50	1.15
LOC_Os06g09950	No	OsAffx.15341.1.S1_at	Expressed protein	1.16	1.14
LOC_Os06g09960	No	OsAffx.27541.1.S1_at	Expressed protein	2.28	1.04
LOC_Os06g09970	No	OsAffx.4783.1.S1_at	Expressed protein	-2.48	2.02
LOC_Os06g09980	AK111302	Os.54864.2.S1_at	Expressed protein	1.49	1.39
LOC_Os06g09990	No	OsAffx.27542.1.S1_s_at	Expressed protein	-1.07	1.00
LOC_Os06g10000	AK108188	Os.55490.1.S1_at	Expressed protein	-2.34	1.05
LOC_Os06g10010	No	No	Expressed protein		
LOC_Os06g10020	AK109163	Os.56042.1.S1_at	Expressed protein	1.02	1.22
LOC_Os06g10030	No	No	Expressed protein		
LOC_Os06g10040	No	OsAffx.27544.1.A1_at	Expressed protein	1.28	1.06
LOC_Os06g10050	No	No	Expressed protein		
LOC_Os06g10060	No	OsAffx.23916.1.S1_at	Expressed protein	1.37	1.06
LOC_Os06g10070	No	OsAffx.4784.1.S1_at	Expressed protein	1.30	1.03
LOC_Os06g10090	No	No	Expressed protein		
LOC_Os06g10100	AK106373	Os.54648.1.S1_at	Expressed protein	-1.14	1.17
LOC_Os06g10109	AK063905	Os.20998.1.S1_at	Expressed protein	-2.33	1.40

^a^ratio of transcripts in WBPH-infested CJ06 to those in uninfested CJ06; ^b^ratio of transcripts in WBHP-infested TN1 to those in uninfested TN1; Positive and negative numerals indicate the gene expression in the infested leaf sheaths was up-regulated or down-regulated, respectively.

## Discussion

The WBPH *Sogatella furcifera* Horváth is a serious insect pest throughout the rice-growing regions of the world, and it has become one of the major threats to rice crops throughout Asia, damaging plants both through its feeding behavior and as a viral vector [28]. The production of resistant varieties is an ecologically sound approach to prevent WBPH infestations [25]. In an effort to use host-plant defenses, attempts to resolve the genetic basis of WBPH resistance in rice have resulted in the identification of the six primary genes *Wbph1* to *Wbph6 *[11]. Sidhu et al. described new sources of major genes conferring resistance to WBPHs in a population prevalent in northern India [29]. However, information regarding the genetic and molecular mechanisms of WBPH resistance in rice is scarce. The major obstacle is in evaluating the resistance to WBPHs; in addition, WBPH resistance in rice has earned a reputation as being difficult to investigate. Nevertheless, QTL analyses using genetic populations derived from crosses of NILs or CSSLs have proven to be powerful tools for investigating the genetic and molecular basis of such quantitative traits [30-32]. Several successful examples of QTL cloning resulted primarily from the development of the corresponding NILs or CSSLs [33-35]. *Bph14* is the first rice insect resistance gene to be cloned as the result of a map-based cloning approach [36]. *Bph14* encodes a coiled-coil, nucleotide-binding, and leucine rich repeat (CC-NB-LRR) protein and confers resistance to BPHs, another major insect rice pest in Asia.

In this study, we performed QTL mapping for the rice ovicidal response that causes the death of WBPH eggs and finely mapped a major QTL for WL production that was delimited to an approximately 122.1-kb DNA region. Yamasaki et al. [4] identified a major QTL for the ovicidal response in the interval between R1594 and L688 on the short arm of chromosome 6, which also contains the *qWL6* region in our study.

There are two different resistance mechanisms to WBPH in some *japonica* rice, namely ovicidal resistance and sucking-inhibitory resistance [17,25]. Ovicidal resistance gives rise to egg mortality in the watery lesions induced at the oviposition sites, while sucking-inhibitory resistance restricts planthopper feeding and colonization on the rice plants [37]. In on our previous work, we identified the Chinese *japonica* variety CJ06 that has these two independent mechanisms, and subsequently constructed a DH population to examine their performance [38]. In this study, we identified a total of 19 QTLs associated with the WLs and EM on eight chromosomes. The expression of the ovicidal response is somewhat suppressed in the sucking inhibitory variety under natural WBPH infestations in the field, because the strong antixenosis against WBPH females in these lines reduces oviposition rates. Nevertheless, the CSSLs used in the fine mapping could lack the sucking inhibitory antixenotic effects.

Among the identified QTLs, some of the loci have been reported in earlier studies in different populations. The QTLs on the long arms of chromosomes 1, 3, 4, 5, and 6 were reported by Yamasaki [4,8]. The loci associated with the WLs and EM were flanked by R1954 and L688 in a near-isogenic line (NIL) population [4], and the QTLs on chromosomes 2 and 7 were identified by Geethanjali [15]. Some QTLs co-localized with those for BPH resistance, such as the locus on chromosome 4 (RM401–RM6997) described by Huang [39]. Tan et al. [16] also showed that two WBPH-resistance genes in rice share the same loci with those for BPH resistance, suggesting the possibility of common loci conferring resistance to both WBPH and BPH in rice. An analysis of the QTL information for both planthoppers in the same mapping population would help to verify this hypothesis.

It is now known that the responses by rice to BPH feeding are most likely similar to pathogen-defense responses [36,40]. For example, *Bph14* is a member of the CC-NB-LRR disease resistance gene family, and it provides resistance to BPH in a mechanism that is fundamentally similar to defense mechanisms against pathogens that activate a salicylic acid-dependent pathway [36].

In this study, *qWL6* was delimited to a 122.1-kb DNA region which contains 20 open reading frames. Two candidate genes, LOC_Os06g09930 and LOC_Os06g09910, were annotated to encode PPCDC and GPCR, respectively. PPCDC belongs to the lyase family, specifically the carboxy-lyases, and catalyzes the decarboxylation of 4′-phosphopantothenoylcysteine to form 4′-phosphopantotheine. In addition, it can act as an inhibitory subunit of the protein phosphatase Ppz1, which is involved in many cellular processes such as the G1-S phase transition and salt tolerance [41]. GPCRs are found only in eukaryotes and are involved in signal transduction. Interestingly, there were no significant differences in the transcript levels of LOC_Os06g09930 and LOC_Os06g09910 between CJ06 and TN1. Nine other unknown genes also had no significant differences in expression. However, using an Affymetrix microarray, four of the unknown genes in the chromosome region containing *qWL6* were found to be differentially expressed between CJ06 and TN1, and real-time RT-PCR authenticated that their differential expression was induced by WBPH feeding. Whether the mapped *qWL6* gene is a homologous gene of known function or a new gene encoding a protein of unknown function should be determined by a genomic sequence analysis and functional complementation assays.

We examined the differential expression of genes responding to a WBPH infestation between the highly resistant CJ06 variety and the susceptible TN1 variety. The gene expression in CJ06 showed more overall activity than in TN1, irrespective of the presence or absence of WBPHs (Additional file 2: Figure S1), and a higher number of differentially induced genes (up- and down-regulated) were found in CJ06 than in TN1. In addition, the expression of genes related to secondary metabolism, defense, and cell and hormone signaling showed prominent differences between CJ06 and TN1, whether infested WBPHs or not. An Asian rice gall midge infestation also elicited diverse responses in rice that involved the induction of genes in primary metabolism, nutrient metabolism and transport, DNA synthesis, defense, and secondary metabolism [20]. Many genes implicated in defense and stress had higher expression levels in the resistant CJ06 variety, even absent a WBPH infestation, and these genes were found to be even more strongly expressed after feeding by WBPH. The results for genes related to secondary metabolism, cell signaling, translation, and protein turnover were similar.

Some differentially expressed genes were anchored in the identified QTL regions. For example, LOC_Os01g64310 and LOC_Os10g11500 localized to the QTL region for WLs between RM5389 and RM401 on chromosome 1 and the region for EM between RM216 and RM467 on chromosome 10, respectively. Some differentially expressed genes related to secondary metabolism and defense were also anchored in the QTL regions (Additional file 3: Figure S2). However, among the genes that were highly differentially induced, none were located in the *qWL6* interval. In addition, some nonsynonymous nucleotide substitutions were identified between the resistant CJ06 and susceptible TN1 varieties, implying a functional difference in the resulting amino acid sequences may play an important role in WBPH resistance.

These results provide important genetic information for improving rice in that the markers tightly linked to *qWL6* could facilitate the incorporation of ovicidal alleles into rice breeding lines and the selection of plants with the ovicidal response. The WBPH populations on some of the varieties derived from the crosses between the *indica* and *japonica* varieties were six times higher than those on the ovicidal *japonica* variety Reiho [4], demonstrating that *qWL6* is crucial for *japonica* varieties. In future breeding, when transferring useful *indica* genes into *japonica* varieties, the improved lines must retain the *japonica* ovicidal allele at *qWL6* to suppress the proliferation of WBPHs. It would also be feasible to transfer *qWL6* into the non-ovicidal *indica* varieties by marker-assisted selection as a means of suppressing the WBPH population in those cultivated areas. The identification of *qWL6* and the ovicidal QTLs is an initial step facilitating the positional cloning of a gene and QTLs that confer resistance to insect oviposition in rice by utilizing the Indel markers linked to *qWL6* and the ovicidal QTLs as the starting points. This positional cloning would also clarify the molecular and genetic mechanisms of the ovicidal response to WBPH.

## Conclusions

We identified 19 QTLs associated with WLs and EM in two different years, and *qWL6* was identified as a major QTL in the rice response to infestation by WBPH. Based on the CSSLs and residual heterozygous population, *qWL6* was delimited to a 122-kb region on chromosome 6. An Affymetrix microarray analysis showed that the resistant-CJ06 and susceptible-TN1 varieties had different responses to WBPH feeding. In addition, four genes in the 122-kb region of the *qWL6* locus were differentially regulated in CJ06 and TN1 in response to WBPH infestation, suggesting they may be candidate resistance genes. These results will facilitate isolating this important resistance gene and its use in breeding WBPH-resistance rice.

## Methods

### Plant materials

The WBPH-resistant *japonica* CJ06 and WBPH-susceptible *indica* TN1 varieties were used as parents to make hybrids. The anthers from the F1 plants were collected and cultured on the inducing medium SK3. Doubled plants were obtained through natural doubling or by treatment with colchicine [26]. This DH population consisted of 170 lines, 120 of which were used for constructing a linkage map and evaluating the ovicidal response at random. This population has also been used for studies on ligule length and leaffolder resistance [42,43].

### Evaluation of the ovicidal response

The evaluation for WBPH resistance was performed as described by Sogawa [26] with minor modifications. The ovicidal response of the rice leaf sheaths at WBPH oviposition sites leads to necrotic discoloration, and the intensity of necrotic symptoms was visually rated from 0 to 3 based on the following categories: 0, no visible necrotic symptoms; 1, brownish oviposition damage, but no WLs; 2, discontinuous WLs; 3, conspicuous, vertically elongated WLs. An average score below 1.0 was defined as the non-ovicidal response, while scores above 1.0 were regarded as an ovicidal response.

EM was measured by using young plants at the early tillering stage, which were grown individually in disposable plastic cups (7 cm diameter, 9 cm high). Gravid females were individually confined on the upper portion of the leaf sheath of each plant with parafilm sachets (2 cm × 2 cm) and permitted to feed and lay eggs for two days at room temperature (26–30°C). Approximately fifteen plants were used for each line. The EM was calculated by counting the live and dead eggs 5–6 days after oviposition by dissecting the leaf sheath tissues at the oviposition sites. Eggs with reddish eye-spots were recorded as developing live eggs, while white opaque eggs were recorded as dead. A 60% EM was defined as the cut-off value to distinguish resistance from non-resistance.

### Development of chromosome segment substitution lines

CSSLs were developed to obtain a pure genetic background and lessen the effect of other minor QTLs. The plants carrying the TN1 genotype flanking *qWL6* were crossed with CJ06 followed by five successive backcrosses. Simultaneously, the SSR markers RM6176 and RM539 were used to initially identify plants having the TN1 genotype in the backcrossed progeny. A set of 74 uniformly distributed SSR markers (Additional file 4: Table S1) on a previous linkage map [43] were used to select individuals in the BC5F1 families containing as little TN1 DNA in the genetic background as possible.

### DNA extraction and PCR analysis

The preparation of genomic DNA for the large screen to select recombinants was performed as previously described [44]. The refined DNA was extracted from fresh rice leaves with the CTAB method [45]. PCR was performed in 20-μl reactions containing 0.2 mM of each primer; 200 mM dNTP mix; 50 mM KCl; 10 mM Tris–HCl, pH 8.3; 1.5 mM MgCl_2_; 0.1% Triton X-100; and 1 unit of Taq polymerase. The PCR program began with an initial denaturation at 94°C for 5 min, which was followed by 40 cycles of 94°C for 1 min, 55°C for 45 s, and 72°C for 50 s with a final extension at 72°C for 10 min. The PCR products were separated by electrophoresis on 3.0–5.0% (w/v) agarose gels and stained with ethidium bromide.

### Marker development

Primers were designed around *qWL6* on chromosome 6 to distinguish the CJ06 and TN1 alleles (Additional file 4: Table S1). The Indel markers were developed based on the sequence differences between *indica* var. 93–11 and *japonica* var. Nipponbare (http://blast.ncbi.nlm.nih.gov/. nih.gov). The sequences were aligned using the SeqMan program of DNAstar (Gene-Codes) to identify the insertions/deletions, and primers flanking the Indels were designed using the Primer Premier 5.0 program and tested on the parents.

### RNA extraction for the GeneChip analysis

Five CJ06 plants and five TN1 plants at the active tillering stage were transplanted into small separate cages enclosed with white nylon mesh, and approximately 100 gravid WBPH females were put into each cage to infest the plants. After 5 days, the upper portion of the leaf sheaths was sampled, pooled, and stored in liquid nitrogen. The leaf-sheath samples from the uninfested rice plants were collected and used as controls. Total RNA was extracted using the TRIzol® reagent and high quality RNA was sent to CapitalBio Corporation for Affymetrix GeneChip Expression Analysis. A total of four chips were used in the experiment, representing both the infested and uninfested parents, CJ06 and TN1.

### Quantitative real-time RT-PCR

The expression levels of four genes (LOC_Os06g09960, LOC_Os06g09970, LOC_Os06g10000, and LOC_Os06g10109) were analyzed using real-time quantitative RT-PCR as a validation of the microarray results. The primers designs are listed in Additional file 5: Table S2. The quantitative assay of the transcript abundance was performed with l μl of each cDNA diluted with SYBR Green Master mix (USA) and assayed with an ABI 7900 sequence detection system according to the manufacturer protocol (Applied Biosystems, USA). Actin mRNA was used as an internal control. The relative quantification method (∆∆C_T_) was used to evaluate the relative abundance of the transcripts [46].

### Data analysis and QTL mapping

An analysis of variance of all phenotypic characters was performed with the JMP statistical package, version 7.0 for Windows (SAS Institute Inc., Cary, NC, USA). Interval QTL mapping was performed for the WLs and EM with MapMaker/QTL 1.1 software. The presence of a QTL was defined as an LOD score larger than 2.0. The genetic variance explained by each QTL and the QTL additive effects were calculated, and the identified QTLs were named according to the standard nomenclature [47].

### Availability of supporting data

The data sets supporting the results of this article are available in the qWL6_FineMapping_DataSets repository in http://www.cnrri.org/upload/2014/qWL6_FineMapping_DataSets.rar.

## Abbreviations

BIL: Backcross-inbred line; BPH: Brown planthopper; Bt: *Bacillus thuringiensis*; CC-NB-LRR: Coiled-coil, nucleotide-binding, and leucine rich repeat; CJ06: Chunjiang 06; DH: Doubled haploid; EM: Egg mortality; GPCR: G protein coupled receptor; LOD: Logarithm of Odds; PPCDC: Phosphopantothenoylcysteine decarboxylase; QTL: Quantitative trait locus; RIL: Recombinant inbred line; RT-PCR: Reverse transcription polymerase chain reaction; SSR: Simple sequence repeat; WBPH: Whitebacked planthopper; WL: Watery lesion.

## Competing interests

The authors declare that they have no competing interests.

## Authors’ contributions

YY, YL, JH and LH performed mapping experiment in segregating population. GZ and LG provided DH populations. YY and LW carried out fine-mapping in segregating population. JX and GX carried out GeneChip real-time RT-PCR Analysis. JL, QQ and DZ supervised the research, designed the experiments and were involved in data analysis. YY wrote the manuscript draft. FC, DZ edited and revised the manuscript. All authors read and approved the final manuscript.

## Supplementary Material

Additional file 1 Biological functional classification of the differentially expressed genes between the CJ06 and TN1. Click here for file

Additional file 2: Figure S1 Cluster display of the differentially expressed genes in infested and uninfested CJ06 and TN1 plants. CJ(+/-), ratio of the transcripts in CJ06 plants infested/uninfested with whitebacked planthoppers (WBPHs); TN(+/-), ratio of transcripts in TN1 plants infested/uninfested with WBPHs; CJ+/TN+, ratio of CJ06 transcripts to TN1 transcripts in WBPH-infested plants; CJ-/TN-, ratio of CJ06 transcripts to TN1 transcripts in uninfested plants. A 3-fold or more difference in expression was used as the criterion. Click here for file

Additional file 3: Figure S2 Differentially expressed genes integrated into the quantitative loci intervals. Open and solid arrows indicate QTLs identified in 2006 and 2007, respectively. Click here for file

Additional file 4: Table S1 SSR markers selected to identify the CSSLs. Click here for file

Additional file 5: Table S2 The developed markers used in this study. Click here for file
