# Supplementary figures and images for: Quantitative trait loci identification, fine mapping and gene expression profiling for ovicidal response to whitebacked planthopper (Sogatella furcifera Horvath) in rice (Oryza sativa L.)
Source: BMC Plant Biol. 2014 May 28;14:145. doi: 10.1186/1471-2229-14-145 (PMC4049401; doi:10.1186/1471-2229-14-145)

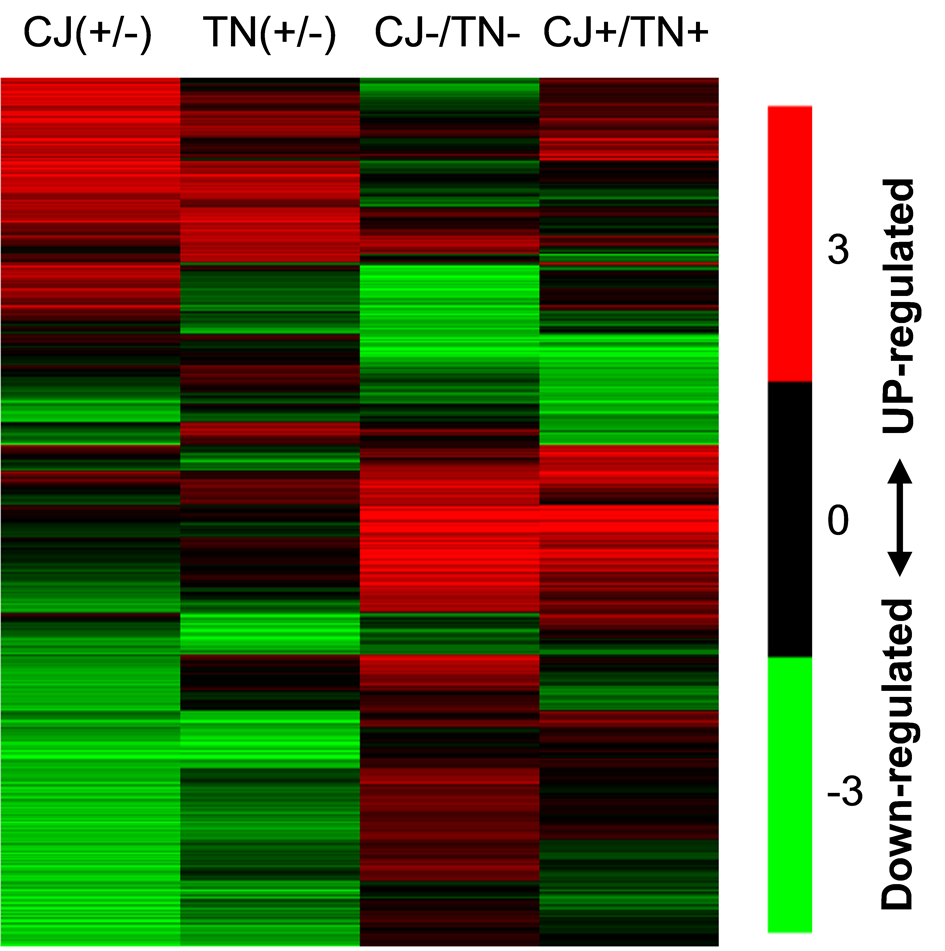

Supplement: Additional file 2: Figure S1 — Cluster display of the differentially expressed genes in infested and uninfested CJ06 and TN1 plants. CJ(+/-), ratio of the transcripts in CJ06 plants infested/uninfested with whitebacked planthoppers (WBPHs); TN(+/-), ratio of transcripts in TN1 plants infested/uninfested with WBPHs; CJ+/TN+, ratio of CJ06 transcripts to TN1 transcripts in WBPH-infested plants; CJ-/TN-, ratio of CJ06 transcripts to TN1 transcripts in uninfested plants. A 3-fold or more difference in expression was used as the criterion. [file 1471-2229-14-145-S2.tiff]

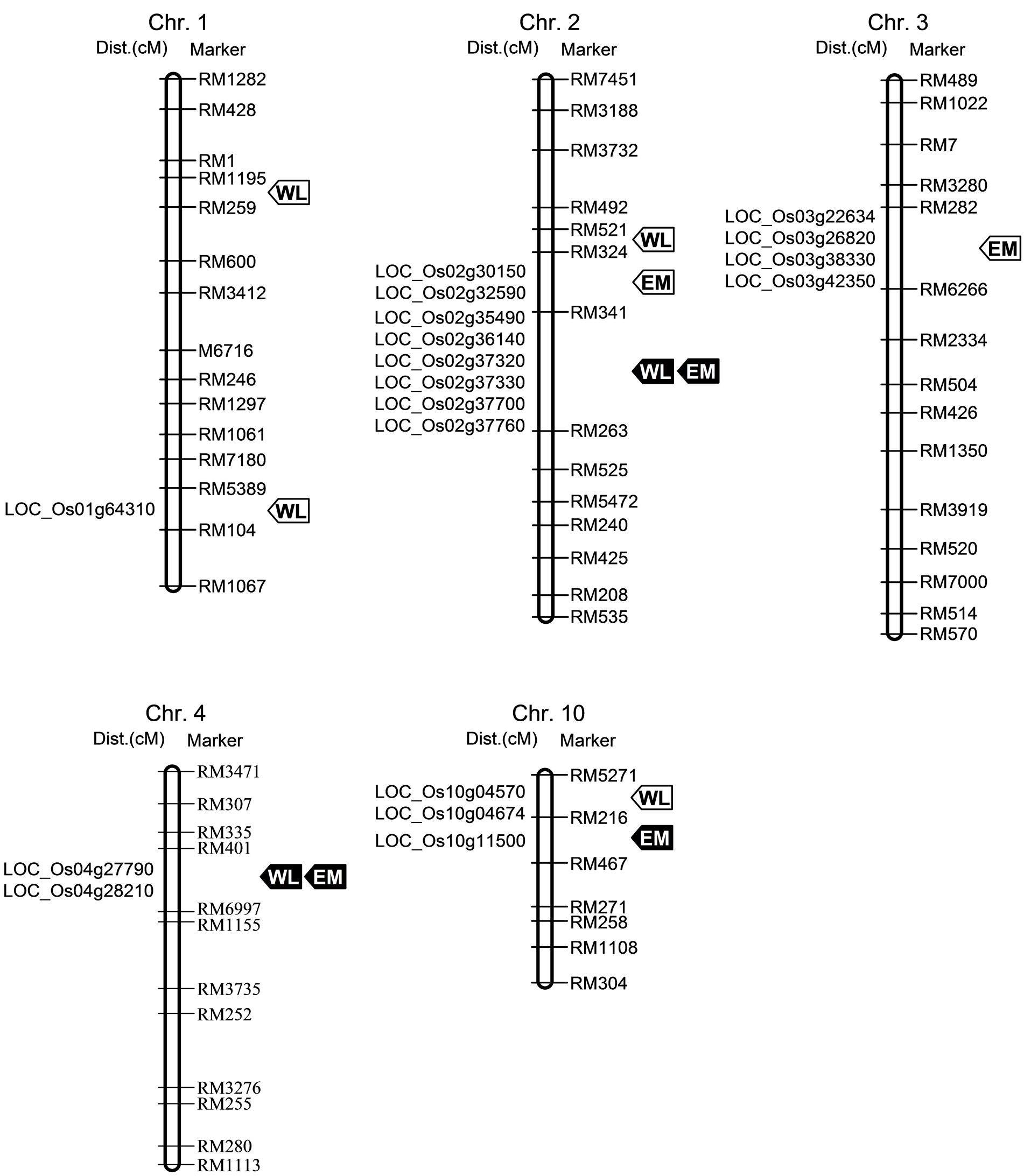

Supplement: Additional file 3: Figure S2 — Differentially expressed genes integrated into the quantitative loci intervals. Open and solid arrows indicate QTLs identified in 2006 and 2007, respectively. [file 1471-2229-14-145-S3.tiff]
