# Supplementary material for: Quantitative trait loci identification, fine mapping and gene expression profiling for ovicidal response to whitebacked planthopper (Sogatella furcifera Horvath) in rice (Oryza sativa L.)
Source: BMC Plant Biol. 2014 May 28;14:145. doi: 10.1186/1471-2229-14-145 (PMC4049401; doi:10.1186/1471-2229-14-145)
Supplement: Additional file 4: Table S1 — SSR markers selected to identify the CSSLs. [file 1471-2229-14-145-S4.doc]

Supplemental table 1. SSR markers selected to identify the CSSLs

| chromosome | SSR markers |
| --- | --- |
| Chro.1 | RM1282, RM5302, RM1195, RM259, RM3412, RM6716, RM246, RM5389, RM104 |
| Chro.2 | RM7451, RM3732, RM492, RM521, RM341, RM263, RM5472, RM425, RM535 |
| Chro.3 | RM489, RM7, RM282, RM6266, RM1350, RM520, RM570 |
| Chro.4 | RM401, RM6997, RM3735, RM3276, RM3306, RM280 |
| Chro.5 | RM440, RM5642, RM6972, RM31 |
| Chro.6 | RM540, RM6176, RM539, RM3, RM162, RM494 |
| Chro.7 | RM8263, RM6449, RM11, RM1279, RM505, RM234 |
| Chro.8 | RM337, RM1376, RM1111, RM331, RM223, RM447, RM3120 |
| Chro.9 | RM5688, RM3700, RM257, RM278, RM1026 |
| Chro.10 | RM5271, RM216, RM467, RM271, RM304 |
| Chro.11 | RM286, RM1812, RM167, RM202, RM3428, RM21 |
| Chro.12 | RM3331, RM1246, RM1103, RM17 |
