# Supplementary material for: Quantitative trait loci identification, fine mapping and gene expression profiling for ovicidal response to whitebacked planthopper (Sogatella furcifera Horvath) in rice (Oryza sativa L.)
Source: BMC Plant Biol. 2014 May 28;14:145. doi: 10.1186/1471-2229-14-145 (PMC4049401; doi:10.1186/1471-2229-14-145)
Supplement: Additional file 5: Table S2 — The developed markers used in this study. [file 1471-2229-14-145-S5.doc]

Supplemental table 2. The developed markers used in this study

| Marker | Forward primer (5'-3') | Reverse primer (5'-3') |
| --- | --- | --- |
| AP4280 | GCATGACTGTATAGTAGCAAGTGTAA | TTTCAAGGGTATGATAAAGGTGAC |
| M1 | ACCCTTCCTTCCTTTCCTCGATC | CTGAGGTCGGCGTCGGTGCA |
| M2 | TTGGTTAGATGGATGAGAATG | CCTGTTAAGGTTAGTGTTTGTTAG |
| M3 | AAAGAGACAAAAGTCCACCCAC | CGCATTTGTATGGGCACAT |
| M4 | ACATGAGACAACGAATCTGGACT | TCCGTCTCAATATATAACACCCTAC |
| M5 | TGAGTTTCCAACCGTCCGTC | ATACACCGTACTCCCTCCGTC |
| M6 | CATTTGAAATACAATTAACTCTCCAC | CTAGTCACCATGTGTCACAAACTG |
| M7 | CATAGGAATCACATCCACCCAT | CCTACTTCGAGCCGTGCCT |
| M8 | TCGTAATCAGGAAAACCAGGA | AGAATAAGAGGACATGACGACTCA |
| AP4687 | AGGAAGCCACATCGAAGTGT | TGAAACGCAAGTGATCTCTCG |
| AP3569 | TCTATTCCAGAAAAAGCCACG | TTAGAATCCCTATAGCAAATGTTG |
| AP4725 | TACCCTTGTCTTCTTCCTCCTT | GGTAAAGGCTAGAGGAGGAGC |
| LOC_Os06g09960 | ATCACCGCCTCTGCCTCCAT | TTCGCCGAGATAGCTTGCCAT |
| LOC_Os06g09970 | TTCTTCGCCGTCGCCACC | TAGACCTACTCGATTCCGCCTGC |
| LOC_Os06g10000 | ACGACCCTGACGGTGTCCCT | ATGATTGGGTTCTTCAAGGCGA |
| LOC_Os06g10109 | TGAAAACAAAAGGTGGAACAGATGA | ACACAACCAGTAGAACTCCTACAGCA |
